# Analyzing public sentiment during national healthcare system failures: A big data approach to emergency department experiences in South Korea

**DOI:** 10.1371/journal.pone.0352142

**Published:** 2026-07-13

**Authors:** Lina Wen, SangYoon Lim

**Affiliations:** 1 School of Economics and Management, Qingdao Agricultural University, Qingdao, China; 2 Seoul National University Bundang Hospital, Seongnam-si, Republic of Korea; National Institutes of Health, University of the Philippines Manila / De La Salle University, PHILIPPINES

## Abstract

**Background:**

The 2024 healthcare crisis in South Korea, characterized by a mass resignation of medical residents and a subsequent “medical void,” has profoundly disrupted the healthcare landscape. This study aims to investigate the emotional responses and behavioral concerns of patients regarding emergency departments (EDs) during this period of systemic instability.

**Methods:**

Utilizing AI-based text mining and sentiment analysis, we analyzed a comprehensive dataset of 1,359,146 unstructured data points harvested from major Korean online platforms between January 1, 2024, and December 31, 2024. The analysis focused on quantifying sentiment polarity and identifying core thematic stressors through keyword frequency and association mapping.

**Results:**

The findings revealed a predominant negative sentiment (45.8%), significantly outweighing positive (32.4%) and neutral (21.9%) responses. Beyond clinical outcomes, specific terms such as “Medical Void,” “Emergency Room Runarounds,” and “Overtreatment” emerged as primary psychological stressors. Temporal and spatial analyses indicated that dissatisfaction spiked during holidays and nighttime, particularly concentrated in metropolitan areas like Seoul, reflecting critical bottlenecks in the emergency care network.

**Conclusions:**

These results suggest that patient dissatisfaction during a national medical crisis transcends service-level issues, reflecting a deep-seated trust deficit and a perceived threat to psychological safety. This study provides a foundational evidence base for developing data-driven crisis communication systems and policy interventions aimed at restoring public trust and enhancing healthcare system resilience during national emergencies.

## 1 Introduction

The COVID-19 pandemic, which began in late 2019, has had a profound negative impact on various sectors worldwide, including the economy, society, culture, and healthcare. To curb the spread of the virus, many nations implemented stringent measures such as travel restrictions, vaccination mandates, social distancing, remote work, and mandatory mask-wearing. While these regulations were necessary for public safety, they simultaneously caused contraction across multiple industries, with the healthcare sector undergoing particularly significant transformations [1].

Prior research indicates that during the pandemic, healthcare consumers often avoided visiting medical institutions due to the fear of infection, leading to a marked decrease in emergency department (ED) utilization [2,3]. Furthermore, issues such as staff shortages and appointment cancellations disrupted the continuity of care, preventing patients from receiving timely medical services [4]. This delay in access has had critical consequences for patients requiring urgent care [5], a problem that has also been documented in South Korea, where pandemic-related disruptions led to significant delays in treatment [6].

Although the pandemic has highlighted the need for solutions to support healthcare consumers in this changing environment [1], patients requiring medical services continue to express dissatisfaction and negative perceptions of the healthcare system [5]. In particular, patients visiting EDs often face overcrowding, unsatisfactory treatment, and privacy concerns, which contribute to negative experiences [7]. Such dissatisfaction not only diminishes patient satisfaction but also undermines the overall quality of medical services, necessitating urgent management strategies [8].

Recently, healthcare consumers have begun to actively search for medical information and share their experiences online to select appropriate providers [9]. This behavior has generated a vast amount of healthcare-related data, making the extraction of key insights from this information a critical task. In particular, analyzing patient sentiment to identify and address negative feedback is essential for improving service quality and achieving organizational performance [10].

Therefore, this study aims to conduct a sentiment analysis of ED experiences using unstructured big data. Specifically, we focus on South Korean healthcare consumers requiring urgent care to analyze negative keywords and associated terms. By identifying the root causes of negative perceptions, such as “medical voids” and “emergency room runarounds,” this study seeks to provide key data for improving ED service quality and enhancing patient safety in the post-pandemic era. In this study, the “medical void” is defined as the systemic clinical service gap resulting from the mass resignation of medical residents. Additionally, “emergency room runarounds” refers to the phenomenon where patients in critical condition are repeatedly rejected by multiple hospitals due to lack of available staff, forcing them to search for care while in transit.

Furthermore, we analyze keywords based on situation, time, and location to identify specific factors hindering service quality.

## 2 Methods

### 2.1 Ethics statement

The present study utilized publicly available, anonymized data from the “Sometrend” big data analytics platform. The research did not involve direct interaction with human subjects, and no personally identifiable information (PII) was collected, processed, or stored. Since the study relied exclusively on existing, publicly accessible data in a manner that does not allow for the identification of individuals, it was determined to be exempt from formal Institutional Review Board (IRB) approval under the applicable ethical guidelines for secondary data analysis.

### 2.2 Data collection

To empirically investigate the sentiments and key issues associated with emergency departments (EDs) in South Korea during the post-pandemic period, this study utilized Sometrend, an AI-based big data analytics platform specialized for processing unstructured text data. Sometrend aggregates data from major search engines and social media channels in Korea, enabling the extraction of keywords, sentiment polarity, and semantic networks from vast amounts of online discourse.

Data collection was conducted for the period from January 1, 2024, to December 31, 2024, capturing the height of the medical void crisis. The search keyword was “emergency room,” and data were harvested from diverse digital channels including blogs, news articles, online communities, and social media platforms such as Instagram and YouTube. A total of 1,359,146 data points were collected for analysis.

### 2.3 Data analysis

The collected unstructured text data were analyzed using text mining techniques, following a two-stage process. Before sentiment classification, the raw text data underwent a rigorous preprocessing pipeline. This included: (1) noise reduction by removing HTML tags, special characters, and URLs; (2) tokenization using a Korean morphological analyzer to identify root words; and (3) deduplication to ensure that identical posts shared across multiple platforms did not skew the frequency counts. This pipeline ensured the purity of the unstructured data before it was fed into the AI engine.

Stage 1: Sentiment Analysis First, a sentiment analysis was performed to quantify healthcare consumers’ emotional responses to ED services. Sentiment analysis classifies text data into positive, negative, or neutral categories, allowing researchers to objectively measure public opinion and satisfaction [11]. With the proliferation of social network services (SNS), patients increasingly express their unfiltered emotions online, making sentiment analysis a critical tool for understanding patient experiences [12]. In this study, we specifically focused on identifying the proportion of negative sentiments to diagnose the current state of ED services in South Korea.

Stage 2: Keyword Frequency and Association Analysis Second, we conducted keyword frequency and association analyses to identify the specific causes of negative sentiment. Keyword frequency analysis extracts the most recurrent terms to determine core themes within a dataset [13], effectively revealing trends and shifts in public interest [14]. Furthermore, association analysis examines the co-occurrence of words to uncover the contextual relationships between key concepts [15]. This approach has been widely adopted in healthcare research to map knowledge structures and track emerging trends [16]. The sentiment analysis was conducted using Sometrend’s hybrid engine, which combines a pre-defined sentiment lexicon with machine learning algorithms specialized for the Korean language. To ensure the reliability of the automated classification, two independent researchers manually validated a random sample of 1,000 data points, achieving an inter-rater reliability (Cohen’s Kappa) of 0.84. Furthermore, all data collection complied with the terms and conditions of the source platforms via Sometrend’s API.

## 3 Results

### 3.1 Data collection overview

The data collection process and summary are presented in Table 1. To investigate sentiments regarding emergency departments (EDs) in South Korea, data were aggregated using the Sometrend platform for the period from January 1, 2024, to December 31, 2024. Data were harvested from diverse digital channels, including major search engines (Naver, Daum), social media (Twitter/X, Instagram, YouTube), blogs, news outlets, and online communities. A total of 1,359,146 data points were collected and utilized for the final analysis

**Table 1:** Summary of data collection.

Category	Description
Platform	Sometrend (AI-based Big Data Platform)
Keyword	Emergency Room
Collection Period	January 2024 – December 2024
Total Volume	1,359,146 data points
Channel Breakdown	Frequency (n) / Percentage (%)
- News (Naver, Daum)	548,661 / 40.4%
- Blogs (Naver, Daum)	265,115 / 19.5%
- Communities (Naver, Daum)	231,842 / 17.1%
- Social Media (X, Instagram, YouTube)	313,528 / 23.1%
Total	1,359,146 / 100.0%

Percentages may not total 100% due to rounding.

### 3.2 Sentiment analysis

**Fig 1:**
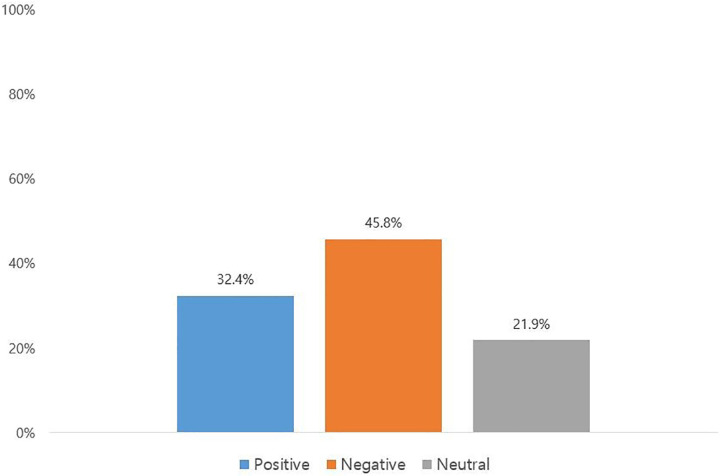
Sentiment analysis results. Sentiment categories were defined based on Sometrend’s standardized emotional lexicon for the Korean language. Overall sentiment distribution regarding EDs: Negative (45.8%), Positive (32.4%), and Neutral (21.9%). Goodness-of-fit test results: χ2 = 117,288.2, df = 2, p < .001. Sentiment categories were defined based on Sometrend’s standardized emotional lexicon.

The distribution of sentiments regarding EDs in South Korea during the study period is illustrated in Fig 1. The results revealed that negative sentiment was the most prevalent, accounting for 46% of the total data. Positive sentiment constituted 32%, while neutral sentiment accounted for 22%. This predominance of negative sentiment indicates a high level of concern or dissatisfaction among healthcare consumers regarding ED services during the healthcare crisis. A Chi-square (χ2) test was performed to determine the statistical significance of these sentiment proportions. The analysis confirmed a significant difference (χ2 = 117,288.2, df = 2, p < .001), proving that negative sentiment was disproportionately dominant during the study period. To address potential platform-specific differences, an additional inferential statistical analysis (Chi-square test of independence) was conducted to compare sentiment distributions between News sources and Social Media. The analysis revealed a significant association between the source type and sentiment polarity (χ2 = 257.81, df = 2, p < .001). Specifically, News content displayed a significantly higher concentration of negative framing (48.2%) regarding the ‘medical void’ compared to user-generated content on Social Media (46.4%), suggesting that media coverage played a key role in amplifying public concern (Table 2).

**Table 2:** Comparison of sentiment distribution between news and social media.

Platform	Positive (n)	Negative (n)	Neutral (n)	Total (n)
News	173,708 (31.7%)	264,532 (48.2%)	110,421 (20.1%)	548,661
Social Media	102,450 (32.7%)	145,547 (46.4%)	65,531 (20.9%)	313,528
Statistical Test	χ2 = 257.81, df = 2, p < .001			

Percentage values represent the proportion within each platform.

### 3.3 Keyword frequency and association analysis

To identify the specific drivers of negative sentiment, the top keywords and their associated terms were analyzed. As shown in Table 3, the most frequent negative keyword was “Pain” (26,596), followed by “Overtreatment” (21,394), “Emergency Situation” (14,879), “Waiting Time” (14,410), and “Medical Void” (13,521). Notably, terms reflecting the 2024 healthcare crisis in Korea, such as “Emergency Room Runarounds” (10,363) and “Medical Personnel Shortage” (9,655), also ranked highly. The associated words for these keywords included descriptors such as “painful,” “unnecessary,” “sudden,” “stressful,” and “concerned,” which highlight the physical and psychological distress experienced by patients during this period.

**Table 3:** Top 10 negative keywords and associated words (raw frequency counts).

Rank	Keyword	Associated Word	Frequency
1	Pain	Painful	26,596
2	Overtreatment	Unnecessary	21,394
3	Emergency Situation	Sudden	14,879
4	Waiting Time	Stressful	14,410
5	Medical Void	Concerned	13,521
6	Emergency Room Runarounds	Severe	10,363
7	Medical Personnel	Insufficient	9,655
8	Pediatric Care	Unable to receive	9,422
9	Suffering	Extreme	8,943
10	ED Operation	Suspended	8,310

Furthermore, the analysis of keywords categorized by time, place, and situation provided contextual insights into ED utilization patterns, as summarized in Table 4. Regarding temporal factors, ED-related searches were most frequent during “Holidays” (39,811), followed by “Nighttime” (33,000) and “Weekends” (28,069), reflecting an increased reliance on emergency services when primary care clinics are typically closed. In terms of location, “Seoul” (35,055) was the most frequently mentioned, followed by “University Hospitals” (30,898) and “Pharmacies” (29,090). Finally, the most common situational contexts were “Emergency Situation” (25,055) and “Death” (17,703), underscoring the critical and often life-threatening nature of ED visits during the study period.

**Table 4:** Top keywords by time, place, and situation (raw frequency counts).

Rank	Time	Frequency	Place	Frequency	Situation	Frequency
1	Holidays	39,811	Seoul	35,055	Emergency	25,055
2	Nighttime	33,000	Univ. Hospital	30,898	Death	17,703
3	Weekends	28,069	Pharmacy	29,090	Pain	12,471
4	Dawn	25,799	General Hospital	28,070	Traffic Accident	8,627
5	Sunday	20,353	Nationwide	16,067	Childbirth	5,257

## 4 Discussion

### 4.1 The prevalence of negative sentiment in the context of a “medical void”

This study empirically identified that negative sentiment (46%) was the dominant emotional response among South Korean healthcare consumers regarding emergency departments (EDs) in the post-pandemic era. This high level of dissatisfaction is significant when compared to general ED satisfaction studies. While EDs inherently involve high-stress situations, the prevalence of keywords such as “medical void” and “emergency room runarounds” suggests that the current negativity is not merely due to clinical outcomes but stems from systemic failures in access. However, these results regarding a “trust deficit” or “threat to psychological safety” should be interpreted as interpreted themes derived from digital discourse rather than direct measurements of psychological states. These findings serve as hypothesis-generating insights into the public’s perception of healthcare resilience. We interpret this as a direct consequence of the 2024 healthcare crisis in South Korea, characterized by the mass resignation of resident doctors and the subsequent reduction in ED capacity [17]. Consistent with previous findings that prolonged waiting times significantly degrade patient satisfaction [18], our results confirm that the structural inability to access care, rather than the care itself, is the primary driver of consumer distress.

### 4.2 Pain and overtreatment: The dual burden of physical and psychological distress

The keyword analysis revealed “Pain” as the most frequent negative keyword, with “Overtreatment” ranking second. This indicates a dual burden on patients: the physical suffering from their condition and the psychological distress caused by distrust in the system. The prominence of “pain” aligns with research identifying pain management as a critical determinant of ED experience [19]. However, in the context of the current medical void, “pain” likely reflects the fear of untreated suffering due to delayed admission, rather than just the symptom itself. Furthermore, the emergence of “overtreatment” and “unnecessary” as top associated words highlights a deep-seated trust deficit. As noted in the literature, defensive medicine or uncertainty in diagnosis often leads to perceptions of overtreatment [20]. In the Korean context, this suggests that patients suspect hospitals of compensating for revenue losses during the crisis through unnecessary procedures, further eroding the patient-provider relationship.

### 4.3 Accessibility gaps: The “seoul-centric” and “holiday” dilemma

The analysis of time and place keywords revealed that ED searches spike during “Holidays” and “Nighttime,” and are heavily concentrated in “Seoul.” This underscores the fragility of the emergency care network during vulnerable times. The concentration of searches in Seoul, despite it having the highest density of medical resources [21], paradoxically indicates that even the best-resourced areas are struggling to meet demand during the crisis. This aligns with prior studies suggesting that the current “emergency room runaround” phenomenon is exacerbating regional disparities and creating bottlenecks even in metropolitan centers [22].

### 4.4 Implications for policy and practice

The findings offer critical implications for health information management and policy:

Crisis Communication Systems: The strong association between “medical void” and negative sentiment necessitates the implementation of real-time, transparent information systems that inform patients of ED capacity and wait times before they travel, reducing the risk of “runarounds.”
Restoring Trust: To address concerns of “overtreatment,” hospitals must prioritize transparent communication regarding diagnostic protocols and costs.
Resource Allocation: Policy interventions should focus on reinforcing ED staffing during high-demand periods (holidays, nights) and decentralizing emergency resources to alleviate the bottleneck in Seoul.

### 4.5 Limitations

This study has limitations. First, demographic variables (age, gender) could not be fully analyzed due to the nature of the aggregated text data. Second, the study did not distinguish between the types of EDs (e.g., regional emergency centers vs. local emergency rooms). Future research should employ panel data to capture individual-level variations in sentiment. Furthermore, as this study aggregated data from diverse platforms including news and SNS, the results may be influenced by ‘media amplification bias.’ Given that news articles constituted 40% of the total volume, journalistic tendencies to focus on systemic failures during national crises may have intensified the overall negative sentiment scores. Future research should integrate qualitative interviews with big data analytics to further validate these interpreted themes.

## 5 Conclusion

The transition to the post-pandemic era in South Korea has been marred by a healthcare crisis that has severely impacted patient experiences in emergency departments [1,6]. This study, utilizing text mining on unstructured big data, reveals that consumer sentiment is overwhelmingly negative, driven by systemic accessibility issues, such as “medical voids” and “emergency room runarounds,” as well as trust-related concerns like “overtreatment” [17,20]. The findings suggest that the current crisis is not merely a logistical challenge but a crisis of information and trust [10]. Healthcare consumers are actively seeking information to navigate a fragmented system, yet they often encounter barriers and uncertainty [9]. Therefore, restoring the quality of ED services requires more than just increasing personnel; it demands a data-driven management strategy that ensures transparent information flow, equitable access during vulnerable times, and the rebuilding of trust between patients and providers [8]. This study provides a foundational evidence base for policymakers to design more resilient emergency medical systems in the endemic era [21].

## Supporting information

S1 File Minimal data set. This Excel file contains the raw frequency counts and sentiment analysis results used in this study.
(XLSX)
